# Recent advances in PLGA micro/nanoparticle delivery systems as novel therapeutic approach for drug-resistant *tuberculosis*

**DOI:** 10.3389/fbioe.2022.941077

**Published:** 2022-07-22

**Authors:** Liqun Shao, Shu Shen, Huan Liu

**Affiliations:** Department of Respiratory, Shenyang Tenth People’s Hospital, Shenyang Chest Hospital, Shenyang, China

**Keywords:** PLGA microparticles, PLGA nanoparticles, drug-resistant *tuberculosis*, *Mycobacterium tuberculosis*, combination therapy, inhalable therapy

## Abstract

*Tuberculosis* is a severe infectious disease caused by *Mycobacterium tuberculosis* and is a significant public health concern globally. The World Health Organization (WHO) recommends a combination regimen of several drugs, such as rifampicin (RIF), isoniazid (INH), pyrazinamide (PZA), and ethambutol (ETB), to treat t*uberculosis*. However, these drugs have low plasma concentrations after oral administration and require multiple high doses, which may lead to the occurrence and development of drug-resistant *tuberculosis*. Micro/Nanotechnology drug delivery systems have considerable potential in treating drug-resistant *tuberculosis*, allowing the sustained release of the drug and delivery of the drug to a specific target. These system properties could improve drug bioavailability, reduce the dose and frequency of administration, and solve the problem of non-adherence to the prescribed therapy. This study systematically reviewed the recent advances in PLGA micro/nanoparticle delivery systems as a novel therapeutic approach for drug-resistant *tuberculosis*.

## Graphical Abstract

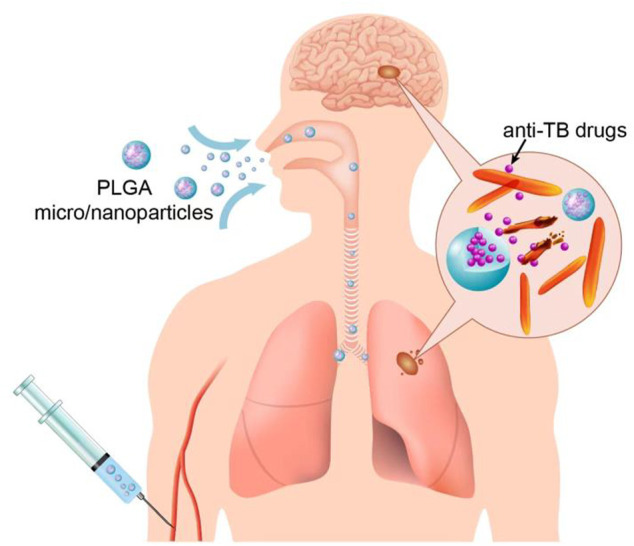

## Introduction

**FIGURE 1:**
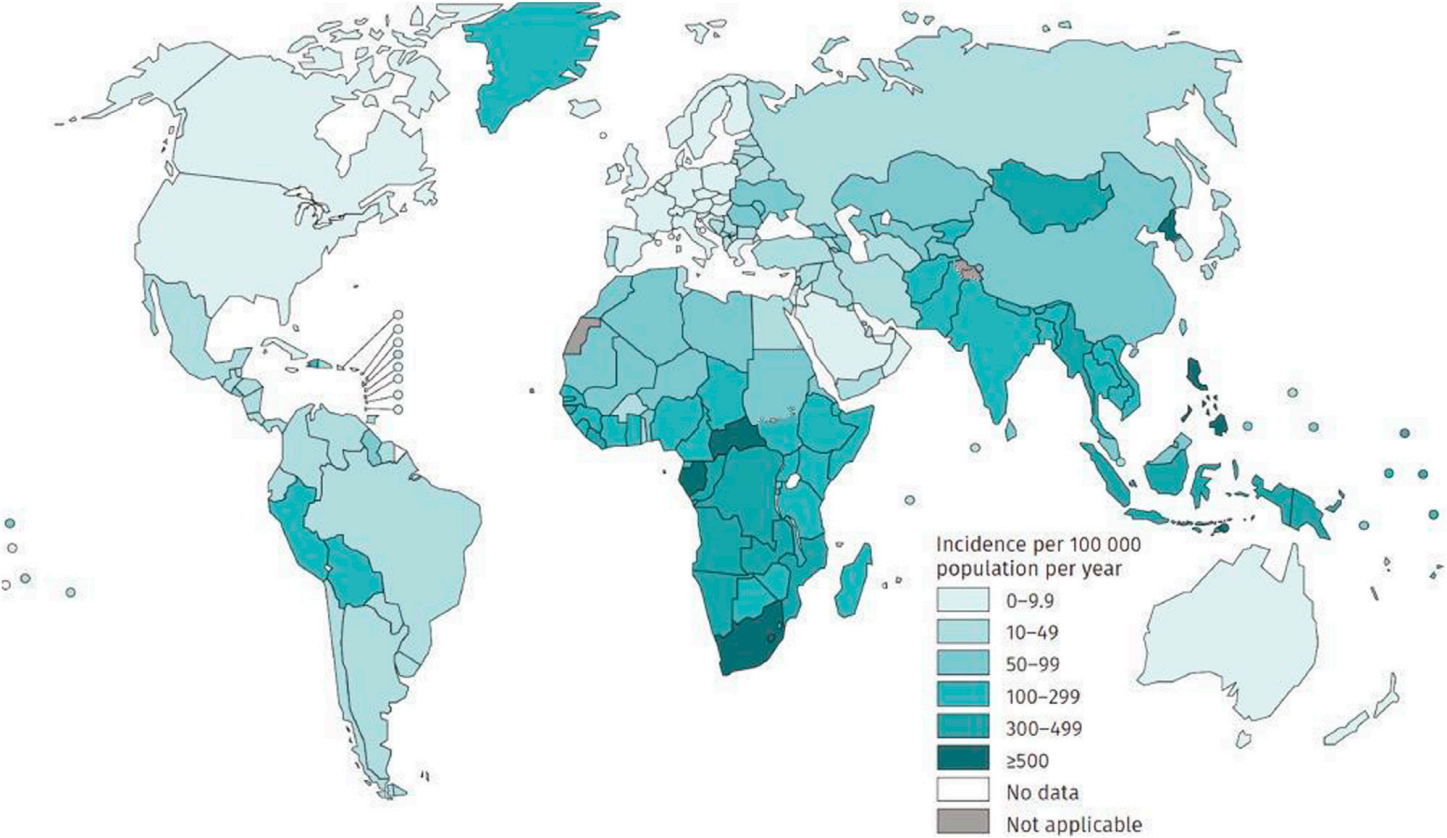
Estimated *tuberculosis* incidence rates, 2020 (excerpted from Global Tuberculosis Report, 2021; World Health Organization, Geneva).

**FIGURE 2:**
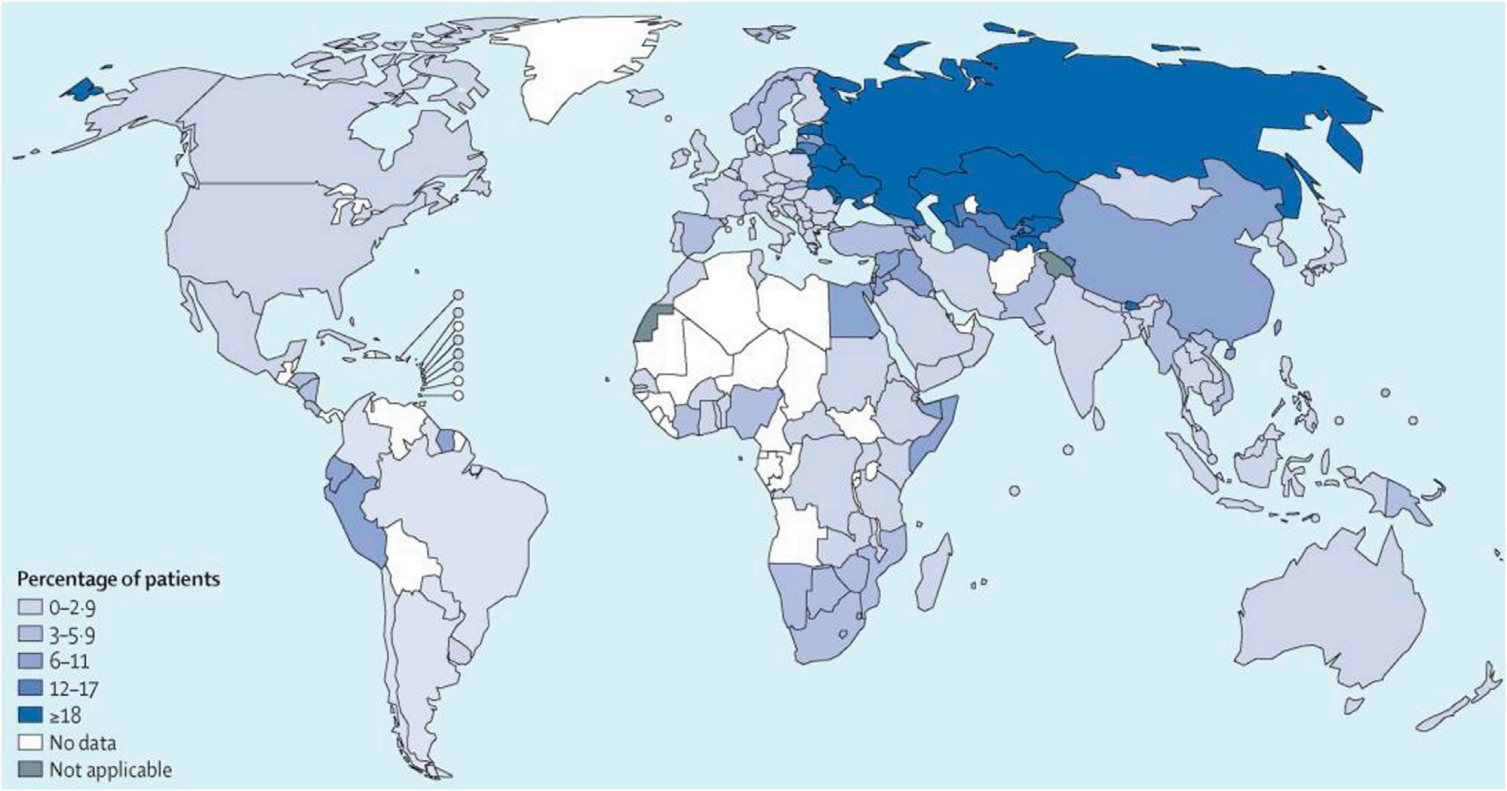
Percentages of patients with *multidrug-resistant tuberculosis* globally. Reproduced with permission from Lange et al. (2019). Copyright © 2019 Elsevier Ltd.

Human *tuberculosis* (TB) is one of the top 10 causes of death globally and the leading cause of death from a single infectious disease (Reid et al., 2019), which has become a global public health emergency (Figure 1). World Health Organization (WHO) estimates that approximately 9.9 million cases have contracted *tuberculosis* worldwide in 2020 (World Health Organization, 2021a), and there were 1.2 million deaths from *tuberculosis* in 2019 (World Health Organization, 2020). Pulmonary *tuberculosis* is the typical manifestation of *tuberculosis*, accounting for approximately 80% of *tuberculosis* cases (World Health Organization, 2020). WHO recommends a 2-month intensive regimen followed by an additional four to 6 months continuation regimen for *tuberculosis* treatment (Table 1). During the intensive treatment phase, patients are administered four anti-TB drugs daily, including rifampicin (RIF), isoniazid (INH), pyrazinamide (PZA), and ethambutol (ETB), while the continuation treatment phase requires daily RIF and INH (World Health Organization, 2010). Usually, poor patient adherence occurs under this administration, resulting in *drug-resistant tuberculosis* (DR-TB), including *multidrug-resistant tuberculosis* (MDR-TB) or *extensively drug-resistant tuberculosis* (XDR-TB) (World Health Organization, 2021b). *Drug-resistant tuberculosis* has become a significant public health concern in many countries (Figure 2). In recent years, the number of patients with *drug-resistant tuberculosis* has steadily increased globally (Lange et al., 2019). According to the WHO Tuberculosis Report 2019, there were nearly 5,00,000 new cases of rifampicin-resistant *tuberculosis* in the world in 2018, of which 78% were MDR-TB (World Health Organization, 2019a). Effectively controlling the epidemic of *drug-resistant tuberculosis* is still one of the significant challenges in the public health field of the world.

**TABLE 1:** Regimen for treatment for *tuberculosis*.

	Under 50 kg	Over 50 kg
Intensive phase (2 months)		
RIF/INH/PYZ/ETB	4 tablets	5 tablets
Combination tablet 120/60/300/200 mg daily, 5 days per week		
Continuation phase (4–6 months)		
RIF/INH		
Combination tablet 150/100 mg	3 tablets	—
Combination tablet 300/150 mg	—	2 tablets

Various treatment approaches have been developed to combat this global emergence of *drug-resistant tuberculosis* (Patil et al., 2018). Recently, drug delivery methods which can deliver anti-tuberculosis drugs to specific sites in a controlled manner have received much attention. To make the anti-tuberculosis drug continuously released at the lesion site, the sustained-release system uses degradable polymers as the carrier comes into being, where the drug can be released for an extended period *via* a membrane or matrix-controlled diffusion (Kumar and Aeila, 2019; Jana et al., 2021). Such systems can reduce dose and frequency of administration to enhance patient compliance with treatment and control the distribution of drugs in tissues and the clearance rate in the blood to minimize the risk of toxicity and other side effects. The development of these advanced drug delivery systems provides an excellent alternative to addressing treatment failure due to patient non-adherence. Several micro/nanoparticles sized sustained-release systems have been designed for drug encapsulation to treat *tuberculosis* (Table 2).

**TABLE 2:** Several micro/nanoparticles sized sustained-release systems for drug encapsulation to treat *tuberculosis*.

Type	Carrier	Drug	Size	Results	References
Microspheres	PLGA/PLA	Rapamycin without/with isoniazid and rifabutin	0.7–4.7 μm	Lung macrophages were better targeted when microsphere-based	Gupta et al. (2016)
				Nanoparticles were used.	
Nanocapsules	Lipid	Tilmicosin	85–186 nm	Tilmicosin-loaded Lipid lipid-core nanocapsules	Al-Qushawi et al. (2016)
				Suggest more efficient treatment in comparison to the conventional Tilmicosin.	
Nanoparticles	Lipid	Rifampicin	315 nm	The mannosylated Nanostructured lipid carriers (NLCS) showed efficient uptake by bone marrow derived macrophages. Further, rifampicin-loaded mannosylatedNLCS were more efficient in reducing mycobacteria’s intracellular growth.	Vieira et al. (2017)
Microspheres	Polyamidoamine dendrimers	Rifampicin	∼6 μm	The formulations could maintain drug plasma concentration above the minimal inhibitory concentration (mic) of an antibiotic for a more extended period	Rajabnezhad et al. (2016)
Nanoparticles	Graphene oxide	Ethambutol	59 nm	Sustained release of the drug resulted in better bioavailability. In addition, the designed formulation demonstrated high biocompatibility with mouse fibroblast cells.	Saifullah et al. (2017)
Micelles	PEG-PLA	Isoniazid/rifampicin	187.9 nm	Loaded micelles are less haemolytic and have lower MIC values for Mtb compared to free drug	Rani et al. (2018)
Nanocapsules	Chitosan	Bedaquiline	328 nm	The *in vitro* antimicrobial activity of the drug against TB strain H37Rv was still as effective as the free drug. Moreover, no cytotoxic effect on A549, hepg2 and THP-1 cell lines of bedaquiline-loaded nanocarriers was observed at the concentration needed to kill the bacteria.	De Matteis et al. (2018)
Micelles	Amphiphilic block copolypeptide	Bedaquiline	∼250 nm	The encapsulated bedaquiline shows increased *in vitro* activity against *Mycobacterium tuberculosis* compared to free bedaquiline.	Soria-Carrera et al. (2019)
Nanoparticles	PCL	Ethambutol	280–300 nm	Nanoparticles reduced mycobacterial infection with the same efficacy observed in the case treated with ethambutol alone.	Helal-Neto et al. (2019)
Nanoparticles	Chitosan	Clofazimine	132–184 nm	Clofazimine nanoparticles were found to be 49.5 times superior in inhibition and anti-mycobacterial activity than free clofazimine.	Pawde et al. (2020)
Nanoparticles	Alginate	Rifampicin		The formulation is non-toxic and has no systemic toxicity after oral administration	Thomas et al. (2020)
Nanoparticles	Human serum albumin	Benzothiazinone	169 nm	Human serum albumin nanoparticle formulations demonstrated an enhanced efficacy compared to the unformulated drug in an *M. tuberculosis* infected macrophage model.	Patel et al. (2020)
Nanoparticles	Bovine serum albumin	Rifampicin	232 nm	Rifampicin-loaded bovine serum albumin nanoparticles demonstrated enhanced *in vitro* therapeutic efficacy compared to the free drug	Joshi and Prabhakar, (2021)
Micelles	Soluplus	Rifampicin	∼107 nm	Rifampicin-loaded PMs enhanced (up to 2.5-fold) the *in vitro* drug microbicidal activity in Mtb-infected THP-1 macrophages versus a rifampicin solution	Grotz et al. (2019)
Nanoparticles	Phospholipid complex	Baicalein	∼200 nm	Mucus-penetrative nanoparticles exhibited a higher diffusion rate in mucus, deeper penetration across the mucus layer, enhanced *in vitro* cellular uptake, increased drug distribution in airways, and superior local distribution and bioavailability compared to mucoadhesive nanoparticles.	Dong et al. (2020)

## Poly (lactic-co-glycolic acid) and poly (lactic-co-glycolic acid) micro/nanoparticles

**FIGURE 3:**
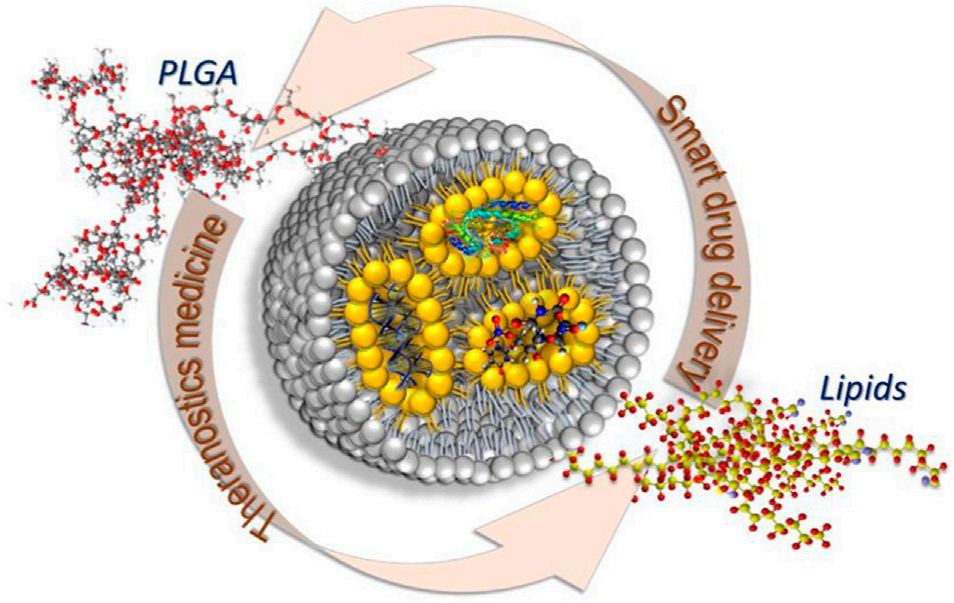
PLGA-lipid hybrid nanocarriers. Reproduced with permission from Ghitman et al. (2020). Copyright © 2020 the Authors.

Poly (lactic-co-glycolic acid) (PLGA) is one of the most successfully developed biodegradable polymers with a wide range of degradation times that can be tuned by its molecular weight and copolymer ratio. PLGA is soluble in common solvents and can be processed into virtually any shape and size. Therefore, PLGA polymers have been primarily tested as delivery vehicles for drugs, proteins, and other macromolecules such as DNA, RNA, and peptides. In addition, the polymers chemical composition and molecular weight, and the physical properties of PLGA nanocarriers, such as size, shape, surface area to volume ratio, etc., can be “tuned” to obtain the desired release profile. With excellent biocompatibility, tunable degradation and release properties and high versatility have been approved for a variety of biomedical applications, PLGA is approved by Food and Drug Administration for human use and has been widely used in sustained-release drug delivery systems (Figure 3) and tissue engineering (Gentile et al., 2014; Mironov et al., 2017; Ding and Zhu, 2018; Lam et al., 2018; Lai et al., 2019; Park et al., 2019; Ghitman et al., 2020; Lagreca et al., 2020; Koerner et al., 2021). PLGA particles are the most widely applied type of particles; the extensive use of PLGA micro/nanoparticle-based drug delivery systems is promising due to their higher efficiency and fewer adverse effects (Chereddy et al., 2016). Table 3 shows the current clinical trials/status of PLGA-based micro/nanoparticles therapy and diagnostics.

**TABLE 3:** Current clinical trials/status of PLGA-based micro/nanoparticles therapy and diagnostics.

Name	Carrier	Drug	Investigated applications	Company	Status	Ref
Pamorelin®	PLGA microsphere	Triptorelin	Prostate cancer	Ipsen Pharmaceuticals	Approved 1986	Jain et al. (2016)
Lupron Depot®	PLGA microsphere	Leuprolide acetate	Prostate cancer, Endometriosis	Takeda-Abbott Products	Approved 1989	Anselmo and itragotri, (2014)
			Central precocious puberty			
Sandostatin Lar®	PLGA microsphere	Octreotide acetate	Endocrinology and Metabolism; Acromegaly	Novartis pharmaceuticals corp	Approved 1998	Zhong et al. (2018)
Trelstar®	PLGA microsphere	Triptorelin pamoate	Prostate cancer	Allergen DM	Approved 2000	Nkanga et al. (2020)
Arestin®	PLGA microsphere	Minocycline HCl	Infectious Diseases	Orapharma Inc.	Approved 2001	Molavi et al. (2020)
			Periodontitis			
Eligard®	PLGA depot	Leuprolide acetate	Prostate cancer	Atrix Laboratories (Tolmar Therapeutics)	Approved 2002	Bobo et al. (2016)
Risperdal Consta®	PLGA microsphere	Risperidone	Neurologic Disorders antipsychotic	Janssen Pharmaceuticals Inc.	Approved 2003	Hrkach and Langer, (2020)
Vivitrol®	PLGA microsphere	Naltrexone	alcohol dependence	Alkermes Inc.	Approved 2006/2010	Dean (2005)
			opioid dependence			
Ozurdex®	PLGA microsphere	Dexamethasone	Corticosteroid	Allergan Inc.	Approved 2009	Cao et al. (2019)
Bydureon®	PLGA microsphere	Exenatide	Type II diabetes	Amylin Pharmaceuticals	Approved 2012	Fineman et al. (2011)
Bydureon Bcise®	PLGA microsphere	Exenatide	Type II diabetes	AstraZeneca AB	Approved 2017	Rentzepis et al. (2018)
Signifor Lar®	PLGA microsphere	Pasireotide pamoate	Acromegaly	Novartis	Approved 2014	McKeage, (2015)
Zilretta®	PLGA microsphere	Triamcinolone	Osteoarthritis	Flexion Therapeutics Inc.	Approved 2017	Chen et al. (2021)
			Other corticosteroid therapy			
Triptodur®	PLGA microsphere	Triptorelin pamoate	Central precocious puberty	Arbor	Approved 2017	Ghitman et al., 2020
Sublocade®	PLGA nanoparticles	Buprenorphine	Moderate to severe addiction to opioid drugs	Indivior Pharmaceuticals	Approved 2017	Chaurasiya et al. (2021)

## Poly (lactic-co-glycolic acid) micro/nanoparticles as novel therapeutic approaches for *drug-resistant tuberculosis*

In recent years, great efforts have been devoted to the development of PLGA Micro/nanoparticle delivery systems for treating *drug-resistant tuberculosis*, and the current research achievements have been listed in Table 4.

**TABLE 4:** Current research achievements of PLGA Micro/nanoparticle delivery systems for treating drug-resistant *tuberculosis*

Carrier	Drug	Method	Size	Results	References
PLGA nanoparticles	Ethionamide	Solvent evaporation	286 nm	There was no significant drug-polymer interaction, and the ethionamide-loaded nanoparticles have no treatment-related toxic effect, which can release sustained for up to 15 days *in vitro*.	Kumar et al. (2011a)
PLGA nanoparticles	Ethionamide	Solvent evaporation	286 nm	When compared to the free drug, the ethionamide-loaded nanoparticles sustained the release of ethionamide for a longer period with significant improvement in pharmacokinetic parameters	Kumar et al. (2011b)
PLGA nanoparticles	Rifapentine	Premix membrane homogenization, solvent evaporation	150 nm	Rifapentine -loaded nps were more effective against *M. tuberculosis* than free RPT.	Liang et al. (2020)
PLGA nanoparticles	Isoniazid, Mycolic acids	Double emulsion solvent evaporation	∼250 nm	The inclusion of mycolic acids in the nanoformulations resulted in their expression on the outer surface and a significant increase in phagocytic uptake of the nanoparticles	Lemmer et al. (2015)
			∼900 nm		
PLGA nanoparticles	Moxifloxacin	Emulsion-evaporation	112 nm	Moxifloxacin-PEG-WSC nps presented striking prolongation in blood circulation, reduced protein binding, and long-drawn-out the blood circulation half-life with resultant reduced liver sequestration vis-à-vis MOX-PLGA nps.	Mustafa et al. (2017)
PLGA nanoparticles	Amikacin, Moxifloxacin	Emulsion evaporation	640 nm	The release of alginate modified PLGA nanoparticles showed slower release in comparison with the non-modified PLGA nanoparticles. Furthermore, the anti-mycobacterial activity of the dually entrapped drug-loaded particles (moxifloxacin and amikacin) was higher compared to single drug-loaded nanoparticle formulations	Abdelghany et al. (2019)
			312–365 nm		
PLGA nanoparticles	Clofazimine	Nanoprecipitation	311 nm	Clofazimine incorporation into the nps was advantageous to reduce drug cytotoxicity. The tfr-binding peptide-functionalized nps showed superior cell interaction and higher Clofazimine permeability compared to the non-functionalized nanoparticles	de Castro et al. (2021)
PLGA microparticles	Gatifloxacin	Solvent evaporation-extraction	40.3 μm, 1.4 μm	Gatifloxaci-loaded PLGA microparticles exhibited high encapsulation efficiency, adequate particle size for pulmonary administration, were rapidly phagocytosed by macrophages, and remained in their interior for at least 48 h	Marcianes et al. (2020)
PLGA microparticles	—	Double emulsion, solvent evaporation	2.2 μm	Drug-free PLGA microparticles could reduce the bacillary viability of THP-1 macrophages	Lawlor et al. (2016)
PLGA microparticles	Rifampicin, All-trans-Retinoic acid	Spray-drying	∼2 μm	ATRA--PLGA microparticles treatments significantly decreased the bacterial burden in the lungs alongside a reduction in pulmonary pathology following just three doses administered intratracheally.	O’Connor et al. (2019)
PLGA microparticles	Isoniazid, Host defence peptides	Double emulsion-solvent evaporation	∼5 μm	The Mucus-penetrating-microparticles dramatically increased (4.1fold) the particle transit through the mucus barrier, which does not adhere to lung mucus, disrupts the bacterial biofilm and provides uniform drug delivery to lungs after pulmonary delivery.	Sharma et al. (2020)
PLGA nanoparticles	Moxifloxacin	Multiple emulsion and solvent evaporation	299.66 nm	After 8 weeks of oral administration of nanoparticles, cfus in the lungs and spleen were reduced.	Vemuri et al. (2016)
PLGA nanoparticles	Econazole	Multiple emulsion and solvent evaporation	561 nm	After 8 weeks of oral administration of nanoparticles, cfus in the lungs and spleen were reduced.	Vemuri et al. (2016)
PLGA nanoparticles	Ethionamide	Multiple emulsion and solvent evaporation	364 nm	After 8 weeks of oral administration of nanoparticles, cfus in the lungs and spleen were reduced.	Vemuri et al. (2016)
PLGA nanoparticles	Thioridazine	Oil-in-water emulsion	211 nm	The thioridazine nanoparticles had no toxicity, and showed a significant therapeutic effect When combined with rifampicin	Vibe et al. (2016)
PLGA nanoparticles	Isoniazid, Moxifloxacin	single emulsion		An enhanced effect of the two drugs was achieved, when they were delivered inside the nanoparticles formulation achieved better antibacterial activity than the free mixture of the drugs	Moin et al. (2016)
PLGA nanoparticles	Levofloxacin, BM2 aptamer	Double emulsification	273.9 nm	BM2- Levofloxacin nanoparticles could gathered accurately in the lesion tissues, and exhibited an excellent therapeutic effect after exposure to ultrasound.	Li et al. (2021)

Kumar et al. (2011a) developed ethionamide-loaded PLGA nanoparticles by solvent evaporation method to achieve prolonged drug release for the treatment of MDR-TB and improve patient compliance. *In vitro* release studies showed that ethionamide was sustainedly released for 15 days in various media. *In vivo* results showed that the ethionamide-loaded PLGA nanoparticles did not show any statistically significant treatment-related effects on weight gain and clinical signs. Likewise, no treatment-related toxic effects were found in haematology, clinical chemistry, and histopathology. The results demonstrate that orally safe ethionamide-loaded PLGA nanoparticles with sustained-release properties offer excellent potential for further preclinical and clinical studies. The authors also investigated ethionamide’s pharmacokinetics and tissue distribution in mice (Kumar et al., 2011b). The sustained release of ethionamide in plasma for 6 days was detected for ethionamide-loaded PLGA nanoparticles compared to 6 h for free ethionamide. Furthermore, ethionamide was detected in organs (lungs, liver and spleen) for up to 5–7 days while maintaining drug levels above the MIC for 5 days, whereas free ethionamide was cleared within 12 h *in vivo* (Kumar et al., 2011b). Ethionamide-loaded PLGA nanoparticles showed significant improvement in pharmacokinetic parameters. Hence, the ethionamide-based PLGA nanoparticles have great potential for reducing the dosing frequency of ethionamide in treating MDR-TB. PLGA-based nanoparticles could also act as a sustained-release delivery vehicle for rifapentine to prolong drug release, alter pharmacokinetics, increase anti-tuberculosis activity, and reduce toxicity, allowing for low dose and frequency (Liang et al., 2020). Future studies on toxicity studies of drug-loaded nanoparticles and the chemotherapeutic potential of ethionamide-loaded nanoparticles against *Mycobacterium tuberculosis* (Mtb) in clinics should be performed.

It is well known that *tuberculosis* is a chronic infectious disease caused by *Mtb* (Khawbung et al., 2021). Resistant strains of *Mtb* cause a significant proportion of *drug-resistant tuberculosis* cases. There is an urgent need to develop new and innovative approaches for treatment. Lemmer et al. (2015) studied mycolic acids as a promising mycobacterial ligand for drug targeting using isoniazid PLGA nanoparticles. The results showed that the phagocytic uptake of mycobacterial acid-coated nanoparticles by mycobacterial-infected macrophages was significantly increased. Therefore, mycolic acid can be further explored as a potential target ligand for various nanoformulations to treat *tuberculosis* effectively.

**FIGURE 4:**
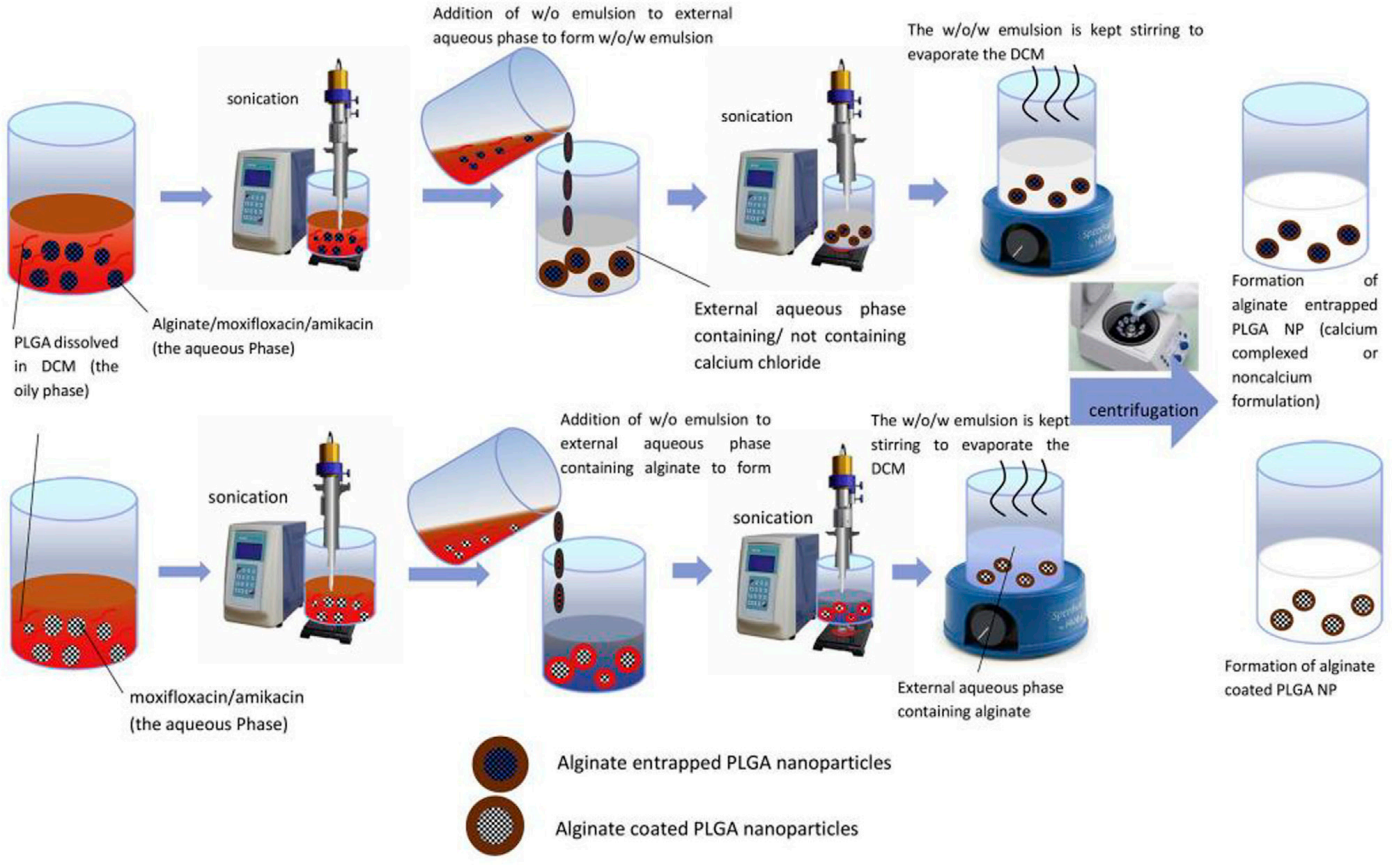
Schematic presentation of **(A)** alginate entrapped PLGA nanoparticles and **(B)** alginate coated PLGA nanoparticles. Reproduced with permission from Abdelghany et al. (2019). Copyright © 2019 Elsevier B. V.

Moxifloxacin (MOX) is an *Mtb* DNA gyrase inhibitor. Due to its strong hydrophilicity, MOX is cleared from the body within 24 h and requires repeated administration, leading to hepatotoxicity and acquisition of MOX-resistant *tuberculosis* associated with use. To overcome the limitations above, Mustafa et al. (2017) developed PLGA nanoparticles to act as an efficient carrier for the controlled delivery of MOX. The authors performed the affixation of polyethylene glycol (PEG) to MOX-PLGA nanoparticles and adsorption of water-soluble chitosan (WSC) to the particle surface to achieve a substantial extension in blood circulation. They investigated the *in vivo* pharmacokinetics and *in vivo* biodistribution after oral administration of the resulting surface-modified nanoparticles (MOX-PEG-WSC NPs), finding that the NPs surface charge was close to neutral +4.76 mV and was significantly affected by the WSC coating. MOX-PEG-WSC NPs significantly prolong blood circulation, reduce protein binding, and prolong blood circulation half-life compared with MOX-PLGA NPs. Therefore, these studies demonstrate that the MOX-PEG-WSC NPs exhibit sustained-release behaviour for controlled drug delivery and prolong the circulation time in the bloodstream for extended periods, thereby minimizing the frequency of dosing and avoiding the occurrence of MOX-resistant *tuberculosis*. Abdelghany et al. (2019) have entrapped amikacin and moxifloxacin into alginate modified-PLGA nanoparticles using two water-oil-water (w/o/w) emulsion strategies (Figure 4), targeting the treatment of MDR. The authors assessed the anti-mycobacterial activity of the resulting PLGA nanoparticles using *Mtb*-infected macrophages. The dually entrapped nanoparticles showed bacterial viability of 0.6% relative to the untreated group, compared to 6.49% for amikacin alone nanoparticles and 3.27% for moxifloxacin alone nanoparticles, revealing an enhanced inhibition of viable bacterial count due to the synergistic effect of moxifloxacin and amikacin in the PLGA nanoparticles (Abdelghany et al., 2019). The amikacin-moxifloxacin alginate entrapped PLGA nanoparticles have the potential to reduce the dose of these drugs, thereby improving patient compliance with treatment and potentially reducing adverse dose-related side effects. However, further *in vivo* studies are urgently required to confirm this prospect.

**FIGURE 5:**
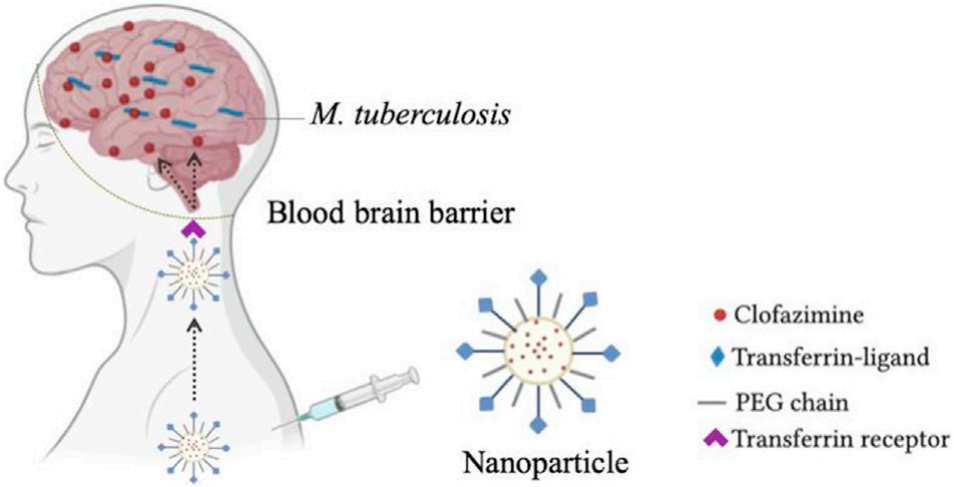
PLGA-PEG nanoparticles loaded with CFZ and functionalized with a transferrin receptor-binding peptide for brain drug delivery. Reproduced with permission from de Castro et al. (2021) Copyright © 2021 Elsevier B. V.

Clofazimine (CFZ) exhibits high activity against multidrug-resistant strains of *Mtb in vitro* (Nugraha et al., 2021; Mashele et al., 2022). However, its poor water solubility and high lipophilicity cause low and erratic drug bioavailability, high plasma protein binding and fatty tissue accumulation, limiting the therapeutic efficacy after oral administration (World Health Organization, 2019b). To solve this problem, de Castro et al. (2021) developed PLGA-PEG nanoparticles loaded with CFZ and functionalized with a transferrin receptor-binding peptide (Figure 5) to develop brain drug delivery to treat the central nervous system *tuberculosis*. *In vitro* studies in brain endothelial hCMEC/D3 cells showed that incorporating CFZ into nanoparticles significantly reduced drug cytotoxicity. TfR-binding peptide-functionalized nanoparticles exhibited better cellular interactions and higher CFZ permeability in hCMEC/D3 cell monolayers than non-functionalized NP controls. The functionalized PLGA-PEG nanoparticles demonstrate suitability for CFZ biological administration, suggested with low plasma protein binding, off-target biodistribution and precise delivery of CFZ towards the brain parenchyma.

**FIGURE 6:**
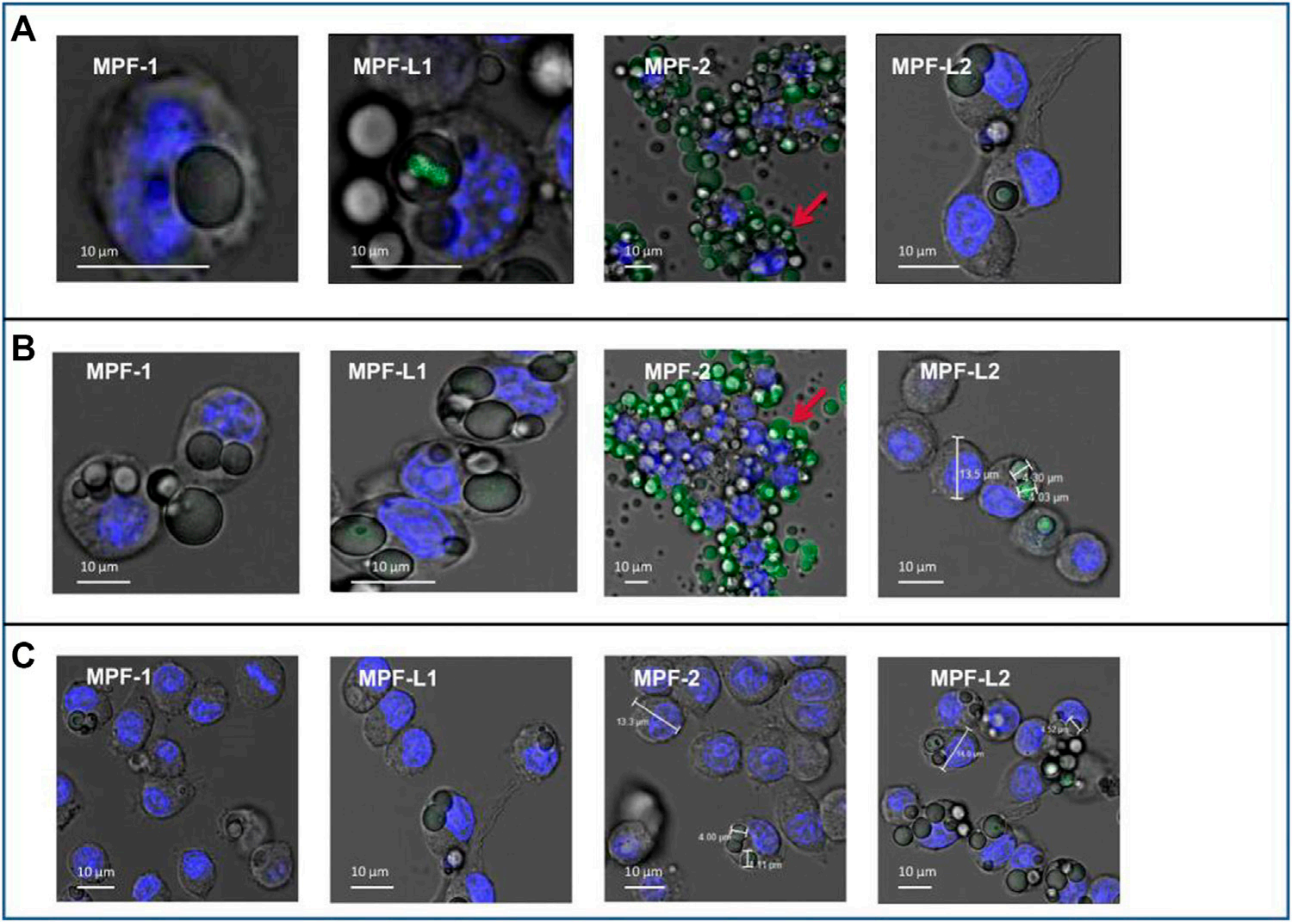
Confocal images of phagocytosis obtained at 3 h **(A)**, 5 h **(B)**, and 24 h **(C)**. MPF-1, fluorescein-loaded PLGA 502 MPs; MPF-2, fluorescein-loaded PLGA 502H MPs; MPF-L1, labrafil-modified fluorescein-loaded PLGA 502 MPs; MPF-L2, labrafil-modified fluorescein-loaded PLGA 502H MPs. Reproduced with permission from Marcianes et al. (2020). Copyright © 2022 Springer Nature Switzerland AG.

*Mtb* can survive and replicate in alveolar macrophages (Ufimtseva et al., 2019), evading host defence mechanisms and developing the latent disease. Considering that phagocytes can localize and internalize foreign substances, such as polymeric particles, this fact could guide particles to the interior of macrophages, leading to an exciting approach to the treatment of intracellular infections affecting the mononuclear phagocytic system. Marcianes et al. (2020) developed a new biodegradable PLGA microparticle for pulmonary administration of gatifloxacin, using labrafil as a surface modifier to actively target alveolar macrophages, thereby allowing gain access of the drug to *Mtb*. Cell phagocytosis was studied in raw 264.7 mouse macrophage cell lines after incubating 3, 5, 24, and 48 h with the microparticles. The results showed that labrafil enhanced the uptake rate of PLGA 502H microparticles by macrophages (Figure 6). Gatifloxacin-loaded PLGA microparticles using PLGA 502H and labrafil exhibited high encapsulation efficiency (89.6% ± 0.2%), rapid phagocytosis by macrophages (3 h), and remained inside the cells for at least 48 h, resulting in a suitable carrier to potentially treat MDR-TB. These results are promising, but regarding future perspectives, further immunogenicity studies of the developed systems and phagocytosis in *Mtb*-infected macrophages should be conducted, and the safety of the formulations should be evaluated in an animal model of *tuberculosis*.

**FIGURE 7:**
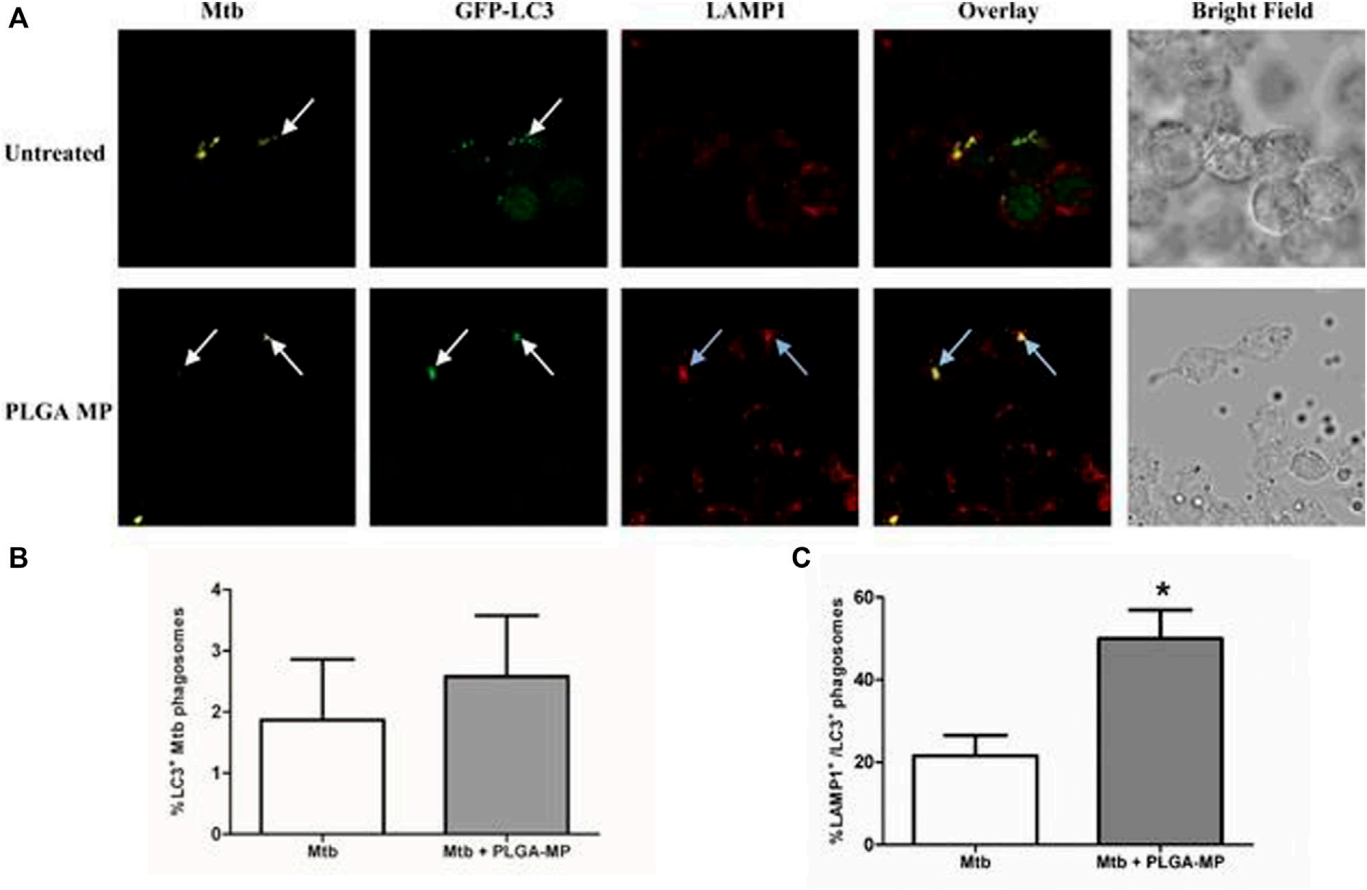
PLGA MPs trigger autophagic flux in Mtb-infected macrophages. **(A)** After a total of 24 h, infected macrophages were fixed and stained with anti-LAMP1_and anti-Mtb antibodies, and then observed by laser scanning confocal microscopy. The proportion of **(B)** LC3 positive Mtb phagosomes and **(C)** LC3 positive phagosomes which were also positive for LAMP1 were counted. Data represent the mean ± SEM of three independent experiments in which more than 100 phagosomes were counted for each condition. *p < 0.05. The white arrows indicate localization of Mtb phagosomes with GFP-LC3, blue arrows indicate co-localization of Mtb, GFP-LC3, and LAMP1. The results shown are the means of three independent experiments. Reproduced with permission from Lawlor et al. (2016). Copyright © 2016 the authors.

Pulmonary drug delivery systems are gaining popularity because of their ability to achieve high drug concentrations at the site of infection and minimize systemic toxicity for the treatment of various lung diseases, including *tuberculosis*. Lawlor et al. (2016) designed PLGA microparticles (MPs) carrying anti-tuberculosis drugs that human alveolar macrophages could successfully take up. They demonstrated how MPs affect macrophage function in *Mtb* infection by treating Mtb H37Ra or H37Rv-infected THP-1 macrophages with MPs. It was found that the activity of NF kappa B was increased in MPs-treated macrophages, although cytokine secretion was unaltered, and the induction of autophagy was demonstrated *via* the Confocal microscopy of immortalized murine bone marrow-derived macrophages expressing GFP-tagged LC3 (Figure 7). Inhibition of caspases did not influence the MP-induced restriction of bacillary growth, however, blockade of NF kappa B or autophagy with pharmacological inhibitors reversed this MP effect on macrophage function. These data support using the inhaled PLGA MP drug delivery system as a vehicle for immunotherapeutic agents and targeted drug delivery, exploiting the generation of inhaled vaccines and inhaled MDR-TB therapeutics.

**FIGURE 8:**
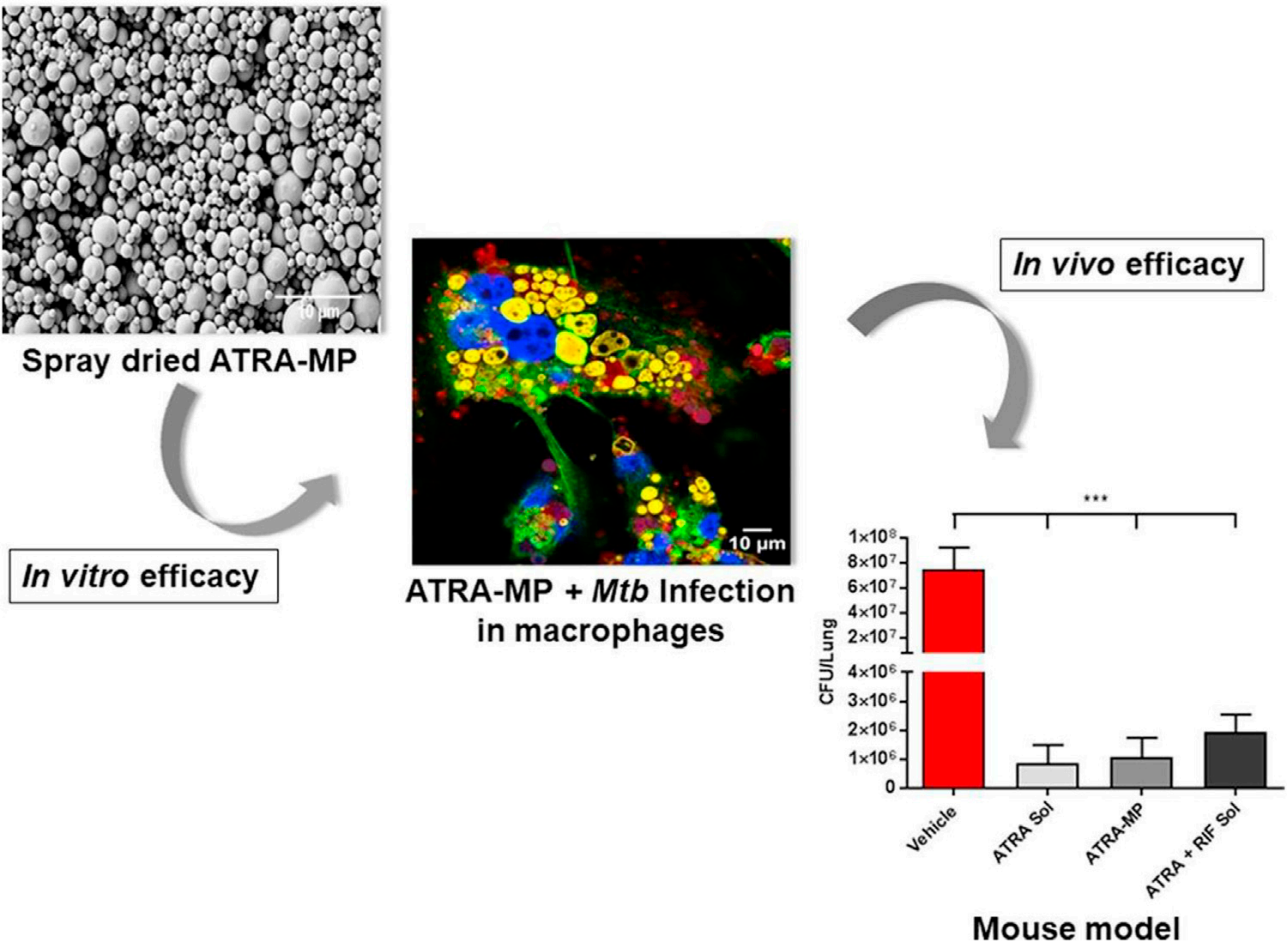
*In vitro* and *in vivo* efficacy of the inhalable PLGA microparticles loaded with trans-Retinoic acid (ATRA). Reproduced with permission from O’Connor et al. (2019). Copyright © 2018 Elsevier B. V.

O’Connor et al. (2019) prepared inhalable PLGA microparticles loaded with trans-Retinoic acid (ATRA) to establish the effect of targeted ATRA treatment on *Mtb* viability. The results showed that 70.5% ± 2.3% of encapsulated ATRA was targeted and delivered to the site of action within the alveolar macrophage, indicating the efficient cellular delivery of ATRA. Furthermore, the results of the BACT/ALERT® system and enumeration of colony-forming units showed a reduction in *Mtb* (H37Ra) growth in THP-1 derived macrophages, confirming the good antibacterial effect of ATRA-loaded PLGA microparticles. The ATRA-loaded PLGA microparticles could also significantly decrease the bacterial burden in the lungs alongside a reduction in pulmonary pathology, as shown in the *in vivo* antibacterial assessment results in Mtb (H37Rv) strain infected BALB/c mice (Figure 8). This study is the first to treat *tuberculosis* with controlled release of ATRA *via* the pulmonary route *in vivo*, providing an alternative to traditional oral supplements that have been ineffective in clinical studies. It provides a new and tried strategy for treating *drug-resistant tuberculosis* and has broad clinical application prospects.

**FIGURE 9:**
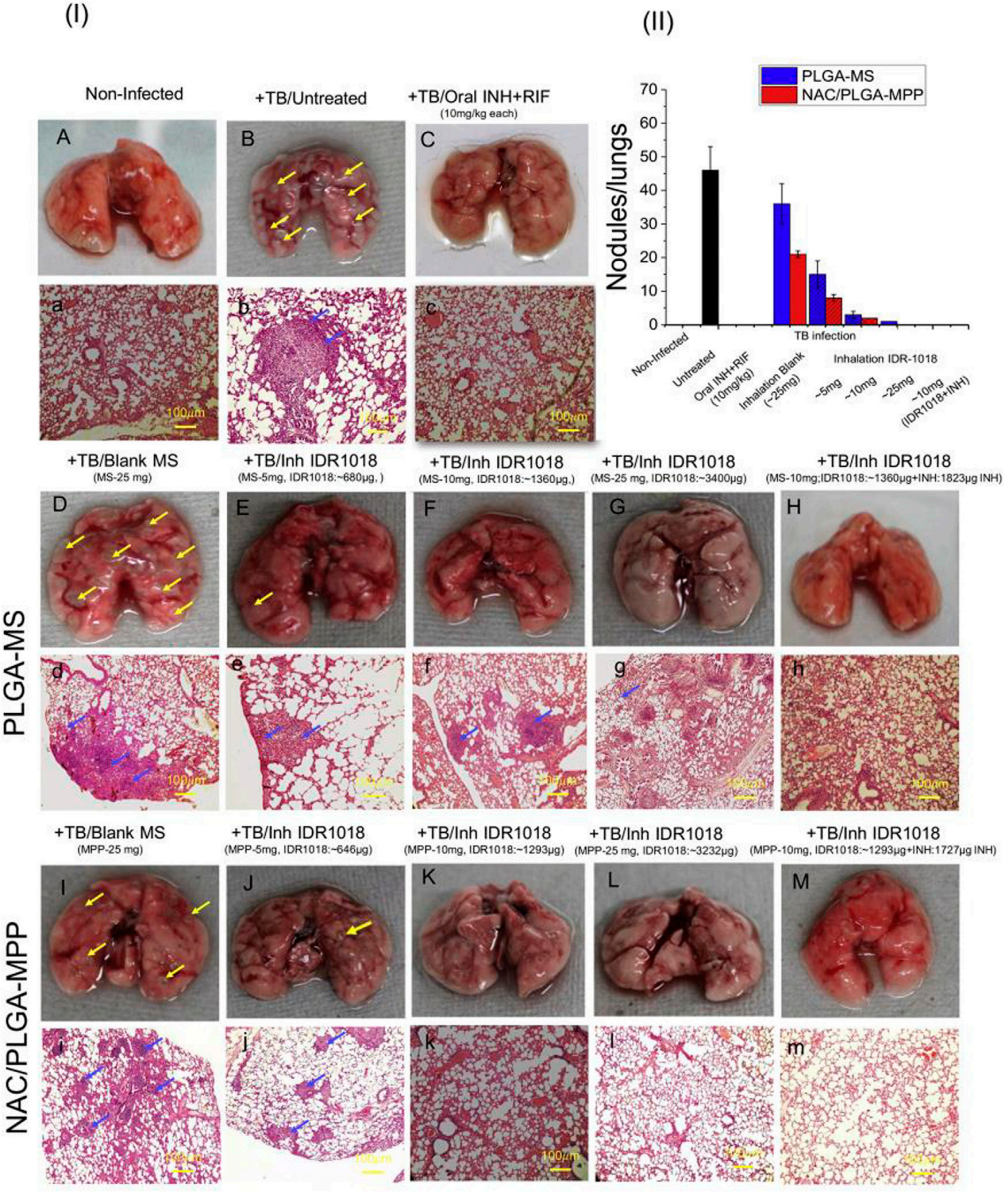
(I) Morphological and histopathological changes in the lungs of mice post Mtb infection and treatment. Representative images showing gross anatomic morphology of whole lungs of Balb/c mice infected with virulent Mtb (H37Rv) and treated with various formulations **(A–I,K–M)** (scale bar: 10 mm). Yellow arrowheads indicate grey-white coloured tubercular nodules (lesion). Histological sections of (**H,E**) stained lungs of normal, infected and treated mice **(a–h,j–m,l)**. Scale bar: 100 μm. Gross pathology photomicrograph showed granulomas (blue arrowheads) in the lungs (*n* = 6 animals/group). Graph show quantitative results of macroscopically detectable tubercular nodules per lung. (II) CFU counting in lungs from mice infected with Mtb. Tissue homogenates of each individual mouse were cultured in agar plates and CFU were counted and averaged. Reproduced with permission from Sharma et al. (2020). Copyright © 2020 Elsevier B. V.

In *tuberculosis* treatment, antibiotics become less effective, and bacteria develop resistance over time due to the formation of some barriers around microorganisms, such as lung mucus and biofilms. Traditional respirable microparticles are mainly trapped in dense, chaotic networks of mucins and are rapidly cleared by mucociliary clearance. Therefore, if the anti-tuberculosis activity of drug-loaded inhalable polymer particles can synergize with the mucus-penetrating and biofilm-disrupting properties, it would be a significant advantage of anti-tuberculosis microparticles, helping to improve the therapeutic effect. Sharma et al. encapsulated IDR-1018 peptide with an anti-tuberculosis drug in N-acetyl cysteine (NAC) decorated porous PLGA microspheres (Sharma et al., 2020), developing Mucus-penetrating-microparticles (NAC/PLGA-MPP). The multiple tracking techniques showed that NAC coating on the porous PLGA microspheres significantly increased (4.1-fold) the passage of particles through the mucus barrier. The designed inhalable NAC/PLGA-MPP does not adhere to lung mucus, does not disrupt bacterial biofilms, and provides uniform drug delivery to the lungs after pulmonary delivery. The activity of the NAC/PLGA-MPP against Mtb in macrophage cultures and in mice model infected with a low-dose bacterial aerosol was evaluated. After 6 weeks of administration of the daily dose, the inhalation of NAC/PLGA-MPP encapsulated with IDR-1018 significantly reduced (*p* < 0.05) bacterial load and inflammation in the lungs in a mouse model of *tuberculosis*. The histopathological results also validate the compelling chemotherapeutic outcome of inhaled formulations (Figure 9). This data supports the potential of using mucus-penetrating inhalable drug delivery systems as a strategy for targeted pulmonary delivery, which may benefit *drug-resistant tuberculosis* treatment.

Combination therapy has been demonstrated as a potentially effective treatment for MDR-TB. Vemuri et al. (2016) encapsulated moxifloxacin (MOX), econazole (ECZ) and ethionamide (ETH) into PLGA nanoparticles to treat the MDR-TB infected mice. Eight weeks of oral administration of individual nanoformulations (PLGA-NP-ECZ/PLGA-NP-MOX/PLGA-NP-ETH) showed limited reduction of CFUs in lungs and spleen, while with 8 doses of a combination of the three nanoformulations (PLGA-NP-ECZ+PLGA-NPMOX+PLGA-NP-ETH) there was a significant reduction in CFUs in lungs as well as in spleen. Corroborating the results with histopathology revealed that the combination of 3-drugs loaded nanoparticles decreased lung congestion to 50%. This is the first report on the potential efficacy of a combination of ECZ, MOX and ETH nanoparticles against MDR-TB. Similar results were reported by Vibe et al. (2016) that when combined with rifampicin nanoparticles, the PLGA nanoparticles loaded with thioridazine gave a modest increase in the killing of both *Mycobacterium* Bovis BCG and *Mtb* in macrophages. The thioridazine nanoparticles showed a significant therapeutic effect combined with rifampicin in the zebrafish, enhancing embryo survival and reducing mycobacterial infection. The results show that thioridazine nanoparticle therapy can improve the antibacterial effect of rifampicin *in vivo*. Moin et al. (2016) also developed a dual drug conjugate PLGA nanoparticle using isoniazid (INH) and moxifloxacin (MOXI) to combat the multi-drug resistance exhibited by mycobacterial species. The drug-conjugate-loaded PLGA nanoparticles are rapidly hydrolyzed into individual parent molecules at the pH of macrophages and function with different mechanisms of action at the same site in macrophages, thereby preventing the development of drug resistance and the development of *tuberculosis*. From the results of the experimental work, it can be concluded that incorporating two or more drugs into *tuberculosis* with the same carrier and target can be an effective strategy against MDR-TB.

**FIGURE 10:**
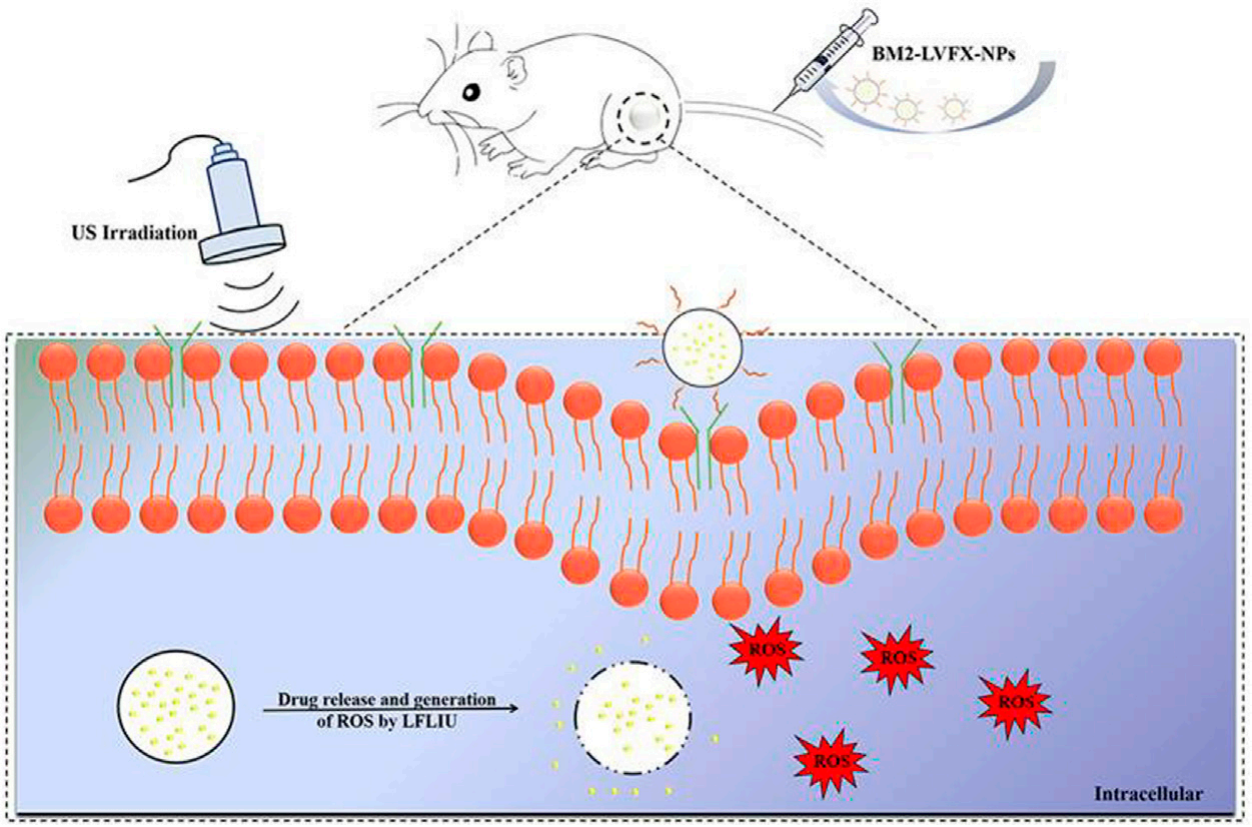
BM2-LVFX-NPs for sonodynamic antimicrobial chemotherapy for BCG infection. Reproduced with permission from Li et al. (2021). Copyright © 2021 the authors.

**FIGURE 11:**
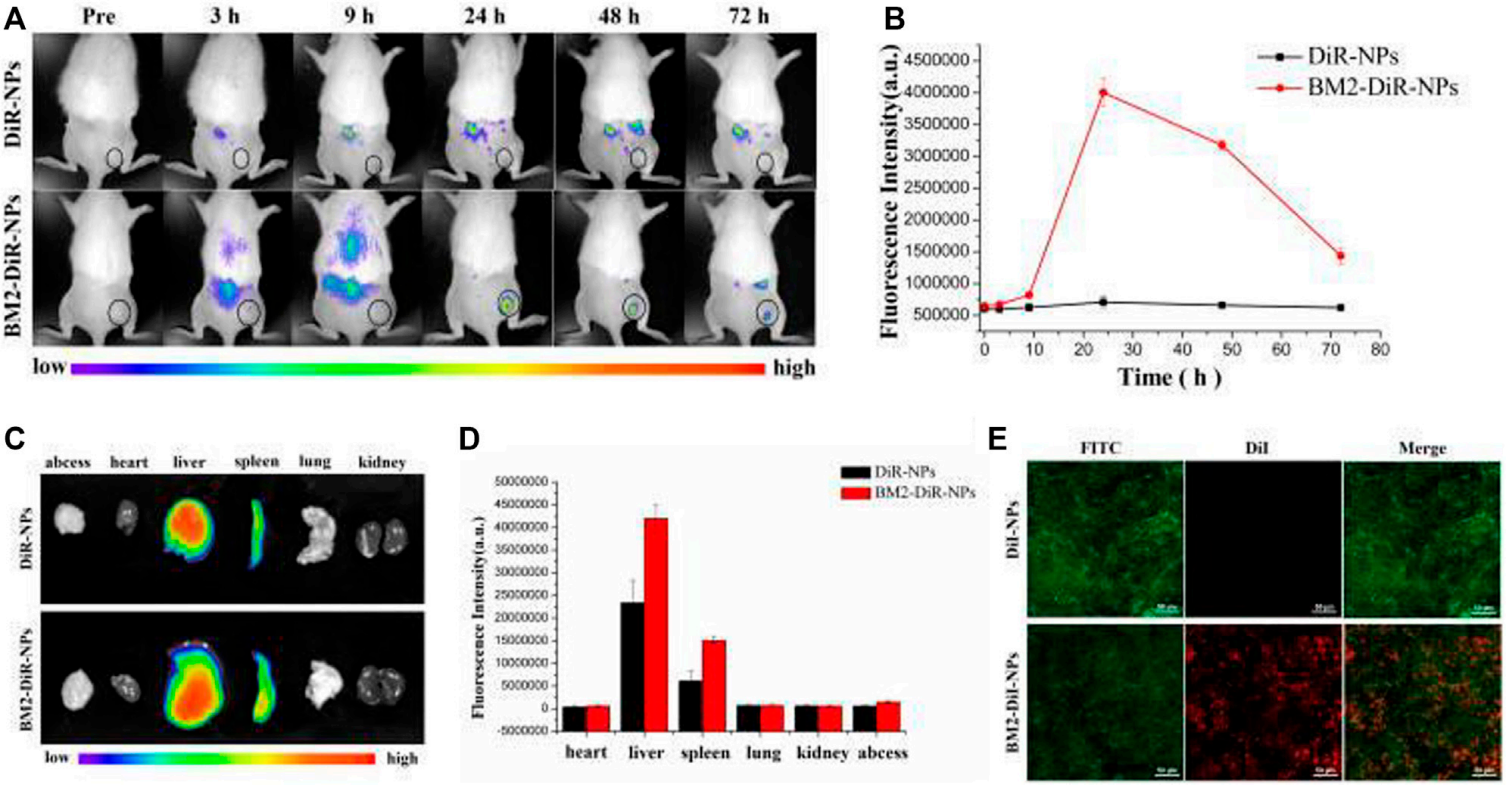
Targeting ability of BM2-modified nanoparticles *in vivo*. **(A)** Fluorescence images of a BCG-infected rat at 3, 9, 24, 48, and 72 h post injection of DiR-labelled nanoparticles. **(B)** Quantitative fluorescence intensity (*n* = 3) of abscess tissue at different time points. **(C)** Biodistribution of DiR-labeled nanoparticles in major organs extracted from rats at 72 h post injection. **(D)** Quantitative analysis of fluorescence intensity (*n* = 3) in major organs. **(E)** CLSM images of Frozen section of abscess tissues at 24 h post-injection of DiR-loaded nanoparticles. The scale bar is 50 μm. Reproduced with permission from Li et al. (2021). Copyright © 2021 the authors.

**FIGURE 12:**
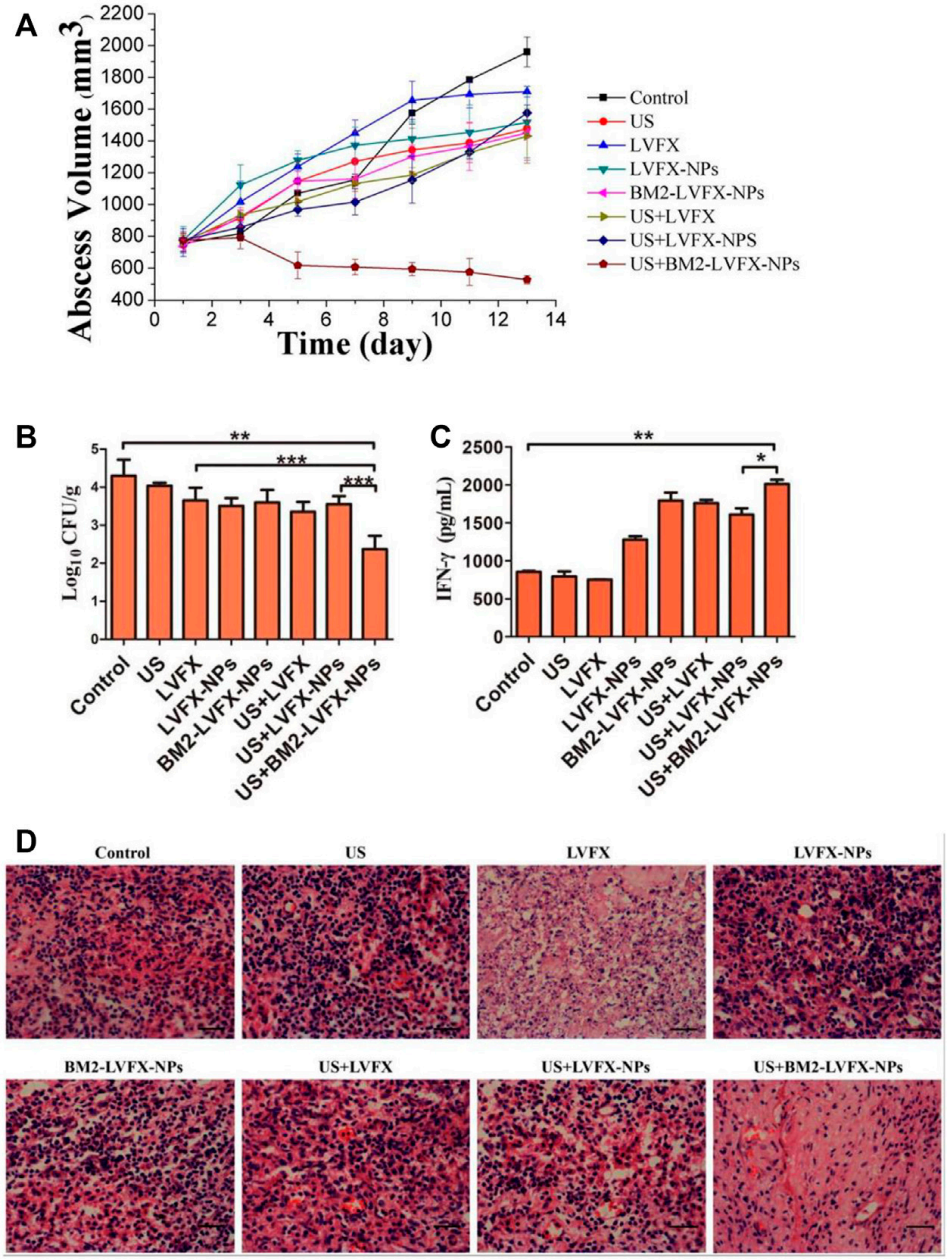
*In vivo* SACT efficacy of BM2-LVFX-NPs combined with ultrasound. **(A)** The time-dependent abscess volume curves of infected rats in each group. **(B)** Colony counting analysis (Log10 CFU) of bacterial cultures from the abscess tissue in the rats after a 14-day treatment. **p < 0.01, ***p < 0.001. **(C)** Serum IFN-γ level of BCG-infected rats on Day 14 after treatment. **(D)** Histopathologic observation of the infected tissues of every group after being treated in various ways. The scale bar is 50 μm. Reproduced with permission from Li et al. (2021). Copyright © 2021 the authors.

Sonodynamic antibacterial chemotherapy (SACT) combined with sonosensitizer-loaded nanoparticles with targeted therapeutic capabilities promises to eliminate bacteria to treat MDR-TB. Li et al. (2021) developed levofloxacin-loaded PLGA-PEG nanoparticles (BM2-LVFX-NPs) with BM2 aptamer conjugated on the surface using cross-linking agents 1-ethyl-3-(3-dimethylaminopropyl) carbodiimide (EDC) and N-hydroxysuccinimide (NHS), to study the antibacterial activity underlying mechanisms of PLGA nanoparticles with targeted therapeutic function against *Bacillus* Calmette-Guérin bacteria (BCG, an *Mtb* model in the presence of ultrasound stimulations (Figure 10). PLGA nanoparticles were specifically recognized BCG *in vitro* and accurately accumulated in the lesion tissue (Figure 11), and the drugs with ultrasound-responsive properties loaded in PLGA nanoparticles were effectively released. Furthermore, PLGA nanoparticles exhibited significant SACT efficiency and higher ROS production levels, resulting in efficient bacterial elimination *in vitro*. Meanwhile, *in vivo* experiments, PLGA nanoparticles showed excellent ultrasound therapeutic effects in a BCG-infected rat model (Figure 12). The results show that PLGA nanoparticles containing levofloxacin can effectively transfer therapeutic drugs into cells and improve the bactericidal effect under ultrasound, which may be a targeted therapy strategy for *Mtb* infection with high biosafety.

## Shortcomings and limitations

Despite recent advances in PLGA micro/nanoparticle drug delivery systems in *tuberculosis* treatment, many issues still need further addressed. First, it is not an easy task to develop a lyophilization process for dry powder of PLGA micro/nanoparticles, and it is necessary to ensure that the lyophilized powder is easy to redisperse, avoiding aggregation and drug precipitation; Second, the total amount of biomaterial and lyoprotector to be inhaled over time should be evaluated in terms ofchronic lung toxicity. The total amount of materials and lyoprotectants, and thus nanomedicines for *tuberculosis* treatment, should be optimized to reduce the daily dose of excipients administered to patients. It is also necessary to emphasize the importance of reproducibility and stability (drug loading, encapsulation efficiency and physicochemical properties) in producing drug-loaded PLGA micro/nanoparticles during mass production. The lack of appropriate and specific regulatory guidelines on characterization, study design, and statistical analysis is a common obstacle to the clinical translation of nanoformulations. There is also a need to optimize shared practices to facilitate the translation of nanotechnology from experimental success to clinical practice. In addition, more *in vivo* data are needed. Only a few studies have shown that the PLGA micro/nanoparticles drug delivery system is effective in preclinical models of infected *tuberculosis*. This is a severe disadvantage as future clinical studies will depend on available preclinical data. The research on PLGA micro/nano drug delivery systems for the treatment of *tuberculosis* is still at an early stage, and more investment and capacity are required to make it possible to obtain commercially available micro/nano formulations.

## Conclusion and prospect

*Drug-resistant tuberculosis* is a significant global disease with high morbidity and mortality and remains a major health problem. As current treatment strategies are inadequate, innovative strategies are needed to improve treatment and reduce mortality. The PLGA micro/nanoparticles can be loaded with single or combined drugs with additive/synergistic effects, allowing lower doses of drugs with reduced side effects while improving antituberculosis efficacy of first- and second-line antituberculosis drugs. PLGA micro/nanoparticles offer great potential for more efficient delivery of *tuberculosis* drugs to lesion sites to improve their efficacy, and the introduction of potent, novel and repurposed drugs will increase the effectiveness of such systems. Although the results obtained so far are too preliminary, it is still believed that PLGA micro/nanoparticles have great potential as a novel therapeutic approach for *drug-resistant tuberculosis* and reduce the risk of *drug-resistant tuberculosis* impacting human health. In order to promote the safe and extensive application of the drug-loaded PLGA micro/nanoparticles in the clinical treatment of *drug-resistant tuberculosis*, the following issues should be paid attention to in the future in-depth research: 1) A reliable animal model is necessarily required to examine the safety of PLGA micro/nanoparticles *in vivo* because the number of *in vivo* studies of these pharmaceutical formulations is minimal and requires careful study for human use; 2) Addressing the processing/manufacturing issues for large scale production at an affordable cost will be a fundamental issue in the future; 3) Furthermore, to further realize the progress and efficient delivery of nanomedicines in the lesion sites for precise drug delivery, active participation and cooperation in the fields of nanotechnology, biomedicine, bioengineering and computer analysis are required to make sure nano-drugs will not face any issues during application in clinical trials.
